# MEF2C and SOCS2 in stemness regulation

**DOI:** 10.18632/oncoscience.279

**Published:** 2015-12-28

**Authors:** Caterina Vitali, Claudio Tripodo, Mario P. Colombo

**Affiliations:** Molecular Immunology Unit, Fondazione IRCCS Istituto Nazionale dei Tumori, Milan, Italy

**Keywords:** SOCS2, hemergency hematopiesis

The physiologic stemness of hematopoietic stem cells (HSC) relies on mechanisms constitutively active under steady state and is fundamental to maintain a lifelong HSC reservoir. On the other side, similar stemness features sustained by partially overlapping molecular circuits, which have recently come into focus, confer aggressive aggressiveness in leukemia clones.

Suppressor of Cytokine Signalling 2 (SOCS2) belongs to the SOCS family, comprising eight members (SOCS1–7 and CIS) with similar structures, which are induced upon JAK/STAT activation and function as negative regulators. Recent evidences have demonstrated that SOCS2 is endowed with immunological functions in differentiated cells but no apparent functions were identified in HSC despite its expression in steady state condition.

Combining analysis of human HSC malignancies and studies on murine HSC under steady state and stress conditions [1], we have recently identified a dual involvement of SOCS2 in the regulation of HSC functions in different contexts and demonstrated a novel regulatory mechanism for SOCS2 expression in HSC.

In mice under hematopoietic stress conditions, such as after 5-Fluorouracil-induced myeloablation, hematopoietic cytokines are rapidly produced to sustain bone marrow (BM) recovery. This event induces activation of the JAK-STAT5 pathway consequently upregulating SOCS2. Such negative feedback loop avoids excessive HSC proliferation and eventually the exhaustion of HSC functions.

This regulatory function of SOCS2 is completely novel, while the JAK-STAT dependency for its expression is common to the regulatory loop involving other SOCS proteins as well as SOCS2 in other contexts [2].

Also, we uncovered SOCS2 involvement in hematopoietic malignancies. High SOCS2 expression characterized the BM of chronic myeloid leukemia (CML) patients and increased along clone progression toward blast crisis. The highest and widespread SOCS2 expression in BM hematopoietic populations was associated with aggressive acute leukemia subsets, namely acute myeloid (AML) and lymphoblastic leukemias (ALL) with MLL rearrangments and BCR/ABL abnormalities. In AML patients, high *SOCS2* was significatively associated with poor prognosis.

In AML and ALL patients, high *SOCS2* expression also positively correlated with a list of genes that significanly overlapped with leukemic stemness gene signatures [3], suggesting that *SOCS2* and hematopoietic stemness can be associated in the context of hematopietic malignancies. Normal HSC and leukemic stem cells (LSC) share some common molecular programs and, conceivably, similar molecular mechanisms could regulate SOCS2 in these populations.

Our analysis of public gene expression profiles of AML and ALL excluded that *SOCS2* expression could be ascribed only to JAK-STAT pathways activation and suggests that alternative STAT-independent molecular programs should be involved. To our knowledge, this is the first indication of STAT-independent regulation of SOCS proteins, raising a question on whether similar regulation might occur for other SOCS family members. Such STAT-independent mechanism might explain the expression of SOCS2 in acute leukemia subsets with MLL rearrangements, which are not strictly associated to constitutive STATs activation.

Computational analysis revealed a novel regulatory network for *SOCS2* reliant on MEF2C, a transcriptional factor already associated to ALL with MLL rearrangement [4]. MEF2C-dependent SOCS2 regulation was confirmed *in vitro* in murine hematopoietic lineage-c-kit^+^Sca1^+^ (LSK) BM precursors upon transduction with *Mef2c*. Such Mef2c-requirement for *Socs2* expression in steady state condition can be overcome by cytokine stimulation in case of hematopoietic stress.

In conclusion SOCS2 appears to take part into a stemness program that is MEF2C-dependent while STAT-independent, a condition than can be inverted in case of stress-induced hematopoiesis. Moreover, the program fronted by MEF2C confers stemness features to the leukemic clones of AML and of ALL with MLL rearrangement, and is detrimental for the patients.

**Figure 1:**
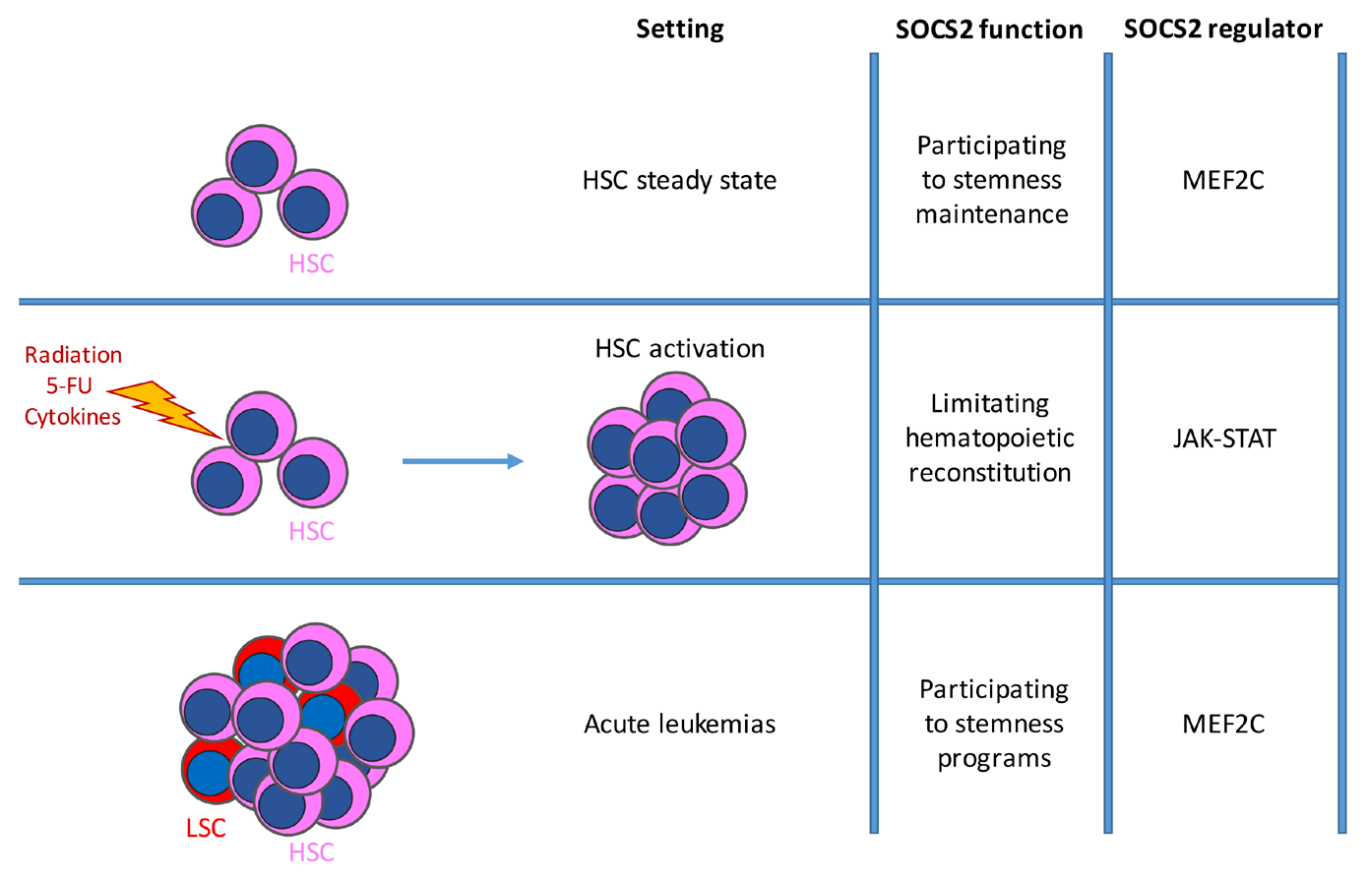
Schematic representation of SOC2 involvement in different hematopoietic settings: normal, steady-state hematopoiesis; myeloablation-induced stress hematopoiesis; malignant hematopoiesis The function of SOCS2 in these settings varies from hematopoietic regulation to stemness program induction according to its control by either STAT-dependent or MEF2C-dependent regulation.
